# Soil Mesofauna Respond to the Upward Expansion of *Deyeuxia purpurea* in the Alpine Tundra of the Changbai Mountains, China

**DOI:** 10.3390/plants8120615

**Published:** 2019-12-17

**Authors:** Yan Tao, Zhongqiang Wang, Chen Ma, Hongshi He, Jiawei Xu, Yinghua Jin, Haixia Wang, Xiaoxue Zheng

**Affiliations:** 1 Key Laboratory of Geographical Processes and Ecological Security in Changbai Mountains, Ministry of Education, School of Geographical Sciences, Northeast Normal University, Changchun 130024, Jilin Province, China; taoy431@nenu.edu.cn (Y.T.); wangzq027@nenu.edu.cn (Z.W.); heh@missouri.edu (H.H.); xujw634@nenu.edu.cn (J.X.); jinyh796@nenu.edu.cn (Y.J.); wanghx885@nenu.edu.cn (H.W.); zhengxx856@nenu.edu.cn (X.Z.); 2 School of Public Administration and Law, Northeast Agricultural University, Harbin 150030, Heilongjiang Province, China; 3 School of Natural Resources, University of Missouri, Columbia, MO 65211, USA

**Keywords:** soil mesofauna, indicator, *Deyeuxia purpurea*, upward expansion, alpine tundra

## Abstract

*Deyeuxia purpurea*, a low-altitude species, has been expanding upwards into alpine tundra, and this upward expansion is causing serious ecological consequences. However, few studies have been performed regarding its effects on soil faunal communities. We examine how the upward expansion of *D. purpurea* affects the abundance, richness, and diversity of soil mesofauna, and evaluate how different taxa of soil mesofauna respond to the upward expansion of *D. purpurea* in the alpine tundra of Changbai Mountains, northeast China. A total of 128 soil mesofaunal samples were collected from four treatments, namely high upward expansion (HU), medium upward expansion (MU), low upward expansion (LU), and native plant habitats (NP). The results revealed that the abundance of soil mesofauna was increased with the rise of *D. purpurea* upward expansion, and the taxonomic composition varied with the different levels of *D. purpurea* upward expansion in the alpine tundra of the Changbai Mountains. No unique taxa were collected in the native plant habitats, and the upward expansion of *D. purpurea* promoted the colonization of predatory invertebrates. Isotomidae and Gamasida responded positively to the herbaceous plant upward expansion, and thus they were considered to be a positive indicator of upward expansion. Hypogastruridae and Enchytraeidae responded relatively negatively, while Oribatida, Actinedida, and Pseudachorutidae had ambivalent responses to the upward expansion. Overall, the abundance of soil mesofauna can indicate the levels of the upward expansion of *D. purpurea*. Soil mesofaunal guild characteristics were altered by the upward expansion. The different taxa of soil mesofauna responded to herbaceous plants’ upward expansion to various degrees. Therefore, this study provide evidence supporting the fact that the abundance of soil mesofauna can indicate the levels of upward expansion of *D. purpurea*, but the responses of soil mesofauna to the upward expansion of *D. purpurea* differ among their taxa.

## 1. Introduction

Alpine tundra, which is characterized by low temperatures and high altitude, is a relatively harsh type of ecological system [1]. Due to strong winds, high solar radiation, and snow cover, its biodiversity is relatively low [2]. Therefore, an alpine tundra ecosystem is unique and fragile, and exhibits sensitive responses to global changes [3]. In order to survive, alpine plants have adapted to the harsh conditions of the tundra by surviving low temperature extremes, changing photosynthesis and respiration rates, avoiding ultraviolet radiation, and defending against desiccation [4,5]. In contrast, it is difficult for low-altitude plants to adapt to alpine tundra enlivenments, and thus risks of upward expansion are relatively low [6]. Nevertheless, some investigation performed over the past decade has shown that low-altitude plants have recently been colonizing in the alpine tundra [7,8,9]. Low-altitude plants expanding upward into alpine tundra is causing serious ecological consequences, mainly including the reduction of native plants and changes to the plant communities’ structure. At present, most studies regarding low-altitude plants expanding upward into alpine tundra have mainly focused on aboveground systems, e.g., evaluating changes of vegetation cover and estimating the plant communities’ diversity, whereas a relatively small number of studies have been performed regarding the effects of low-altitude plants’ upward expansion on belowground ecosystems.

Soil fauna play crucial roles in nutrient cycling and energy flows of belowground ecosystems, and also participate in soil ecosystem services [10]. Due to the narrow geographic scope of their activities, as well as their poor ability to migrate, soil fauna is sensitive to changes in soil environments [11]. Previous studies have shown that if changes to the soil or vegetation occur, and if these exceed the limitations of adaption and regulation, then the survival and reproduction of the soil fauna could be affected, and in turn this could potentially result in the extinction or outbreak of some species. Consequently, soil fauna as an indicator has become a hot topic in the study of belowground ecosystems. Previous research has demonstrated that low-altitude plants expanding upward into alpine tundra can effectively replace the native species by establishing positive plant–soil feedback, thereby significantly altering the soil properties [12,13]. This inevitably affects soil faunal taxonomic composition and distribution patterns. However, there is presently a lack of information regarding the responses of soil fauna to low-altitude plants expanding upward into the alpine tundra.

*Deyeuxia purpurea*, a type of herbaceous plant, is widely distributed in the dwarf birch forests of the Changbai Mountains, where the altitude is comparatively lower than alpine tundra. Since the 1990s, however, *D. purpurea* has been expanding upward into the alpine tundra area [14]. This situation provides a good opportunity for exploring the relationship between herbaceous plants upwardly expanding and soil mesofauna. In order to better understand the responses of soil fauna to low-altitude plants’ upward expansion into the alpine tundra, we selected the tundra of the Changbai Mountains as the experimental site. The soil mesofauna were collected from treatments with different levels of *D. purpurea* upward expansion. Here, we hypothesized that the (H1) upward expansion of *D. purpurea* promotes abundance, richness, and diversity of soil mesofauna, while (H2) different taxa of soil mesofauna respond to the upward expansion of *D. purpurea* to various degrees.

## 2. Results

### 2.1. Taxonomic Composition of the Soil Mesofaunal Communities

In this study, a total of 8340 soil mesofauna, belonging to 30 taxa (families/suborders) and 15 orders, were collected from the four treatments. The dominant taxa were found to be Isotomidae (27.77%), Oribatida (23.50%), Gamasida (14.29%), and Actinedida (12.42%). In addition, Hypogastruridae (7.10%), Pseudachorutidae (7.05%), and Enchytraeidae (2.06%) were considered to be common, together accounting for 16.21% of the total number. In addition, the other 23 taxa were infrequently collected, together accounting for 5.81% of the total number of soil mesofauna.

**Figure 1:**
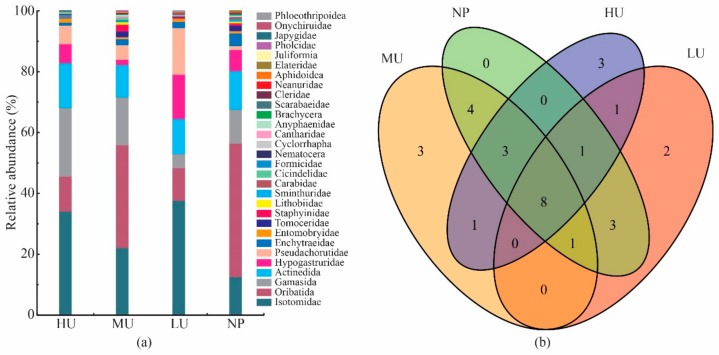
Soil mesofaunal composition in the different treatments. (**a**) Relative abundance of soil mesofauna; (**b**) Venn diagram of the number of shared and unique mesofaunal taxa in different treatments. The shared and unique numbers in the ovals indicate either the unique number of taxa or number of shared taxa in the overlap regions. HU: high level of *D. purpurea* upward expansion; MU: medium level of *D. purpurea* upward expansion; LU: low level of *D. purpurea* upward expansion; NP: native plant habitats.

The taxonomic composition of the soil mesofauna from the different treatments is displayed in Figure 1a. In the high and medium upward expansion treatments and native plant habitats (HU, MU, and NP, respectively), the dominant taxa of soil mesofauna were found to be Isotomidae, Oribatida, Gamasida, and Actinedida. In the low upward expansion treatment (LU), however, Isotomidae (37.78%), Oribatida (10.71%), Actinedida (11.52%), Hypogastruridae (14.55%), and Pseudachorutidae (15.35%) were frequently collected, together accounting for 89.91% of the total number of soil mesofauna. 

Venn diagrams showing the unique and shared taxa are detailed in Figure 1b. The four treatments had eight taxa (Isotomidae, Oribatida, Gamasida, Actinedida, Hypogastruridae, Pseudachorutidae, Enchytraeidae, and Entomobryidae) in common, which in combination contributed to between 40% and 50% of the full set of taxa in each treatment. In the HU treatment, Formicidae, Juliformia, and Japygidae were the unique taxa. Cicindelidae, Aphidoidae, and Pholcidae were recognized only in the MU treatment. Onychiruidae and Phloeothripoidae were found only in the LU treatment. In contrast, no unique taxa were collected in the native plant habitats (NP).

### 2.2. Distribution of Soil Mesofauna

In this study, the distribution characteristics of soil mesofauna were different among the four habitats. The abundances of soil mesofauna are summarized in Table 1 and Table S1. The upward expansion of *D. purpurea* increased soil mesofaunal abundances in the alpine tundra of the Changbai Mountains. A total of 15,625 individuals/m^2^ of soil mesofauna were found in the HU treatment, 14,450 individuals/m^2^ were found in the MU treatment, 12,375 individuals/m^2^ in the LU treatment, and 9675 individuals/m^2^ in the native plant habitats (NP). Among these treatments, the abundances of soil mesofauna in the HU and MU treatments were significantly greater than those in the other treatments (*p* < 0.05). At the same time, the lowest number was found in the native plant (NP) habitats, and it was significantly lower than those in the other treatments (*p* < 0.05).

The richness of soil mesofauna is detailed in Table 1, and it was as follows: MU = NP > HU > LU. The diversity was different among the four treatments, and trends in diversity levels were similar to those for richness (Table 1). The Shannon–Wiener index was greatest in the MU treatment, and it was significantly greater than those in the other treatments (*p* < 0.05). the minimum for the Shannon–Wiener Index was observed in the LU treatments, and there were no significant differences between the HU, LU, and NP (*p* > 0.05).

**Figure 2:**
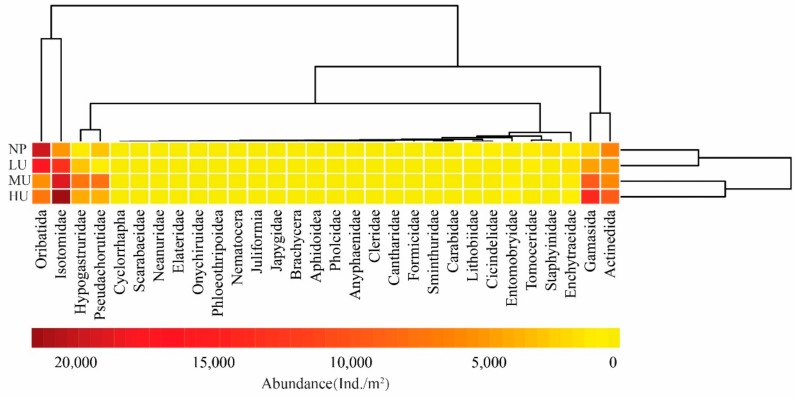
Abundance heatmap of log (*x* + 1) normalized soil mesofauna in the different treatments. A dendrogram of sample sites based on similarity is along the right axis, and a dendrogram of soil arthropod taxa based on similarity is along the upper axis. The colors represent abundance of soil mesofauna. HU: high level of *D. purpurea* upward expansion; MU: medium level of *D. purpurea* upward expansion; LU: low level of *D. purpurea* upward expansion; NP: native plant habitats.

A heat map shows that the four treatments could be divided into two clusters, thus indicating that a major similarity exists between HU and MU. At the same time, it was also revealed that the LU and NP had a high similarity (Figure 2). The soil mesofaunal taxa could be divided into four clusters: Gamasida and Actinedida made up one cluster, Isotomidae and Oribatida respectively composed two more, and the other taxa made up a final cluster. A greater number of Oribatida were found in the LU and native NP, and the majority of Isotomidae and Gamasida were distributed in the HU and MU. Actinedida displayed an evenness throughout all treatments. In addition, the distribution of other soil mesofaunal taxa was relatively rare in the alpine tundra of the Changbai Mountains.

### 2.3. Correlations of Herbaceous Plant Upward Expansion to Soil Mesofauna

The soil properties and plant biomass in each treatment are demonstrated in Table 2. In the HU treatment, the contents of the soil PLFA were significantly greater than in the other treatments (*p* < 0.05). Compared with other treatments, the contents of soil organic matter—total N, total P, and plant biomass—in the MU treatment were all at the highest levels. The soil properties and plant biomass in the LU treatment were all at relatively low levels, and were significantly lower than those in the HU and MU (*p* < 0.05). Furthermore, the amount of soil organic matter and total N in the native plant habitats (NP) was relatively higher.

**Figure 3:**
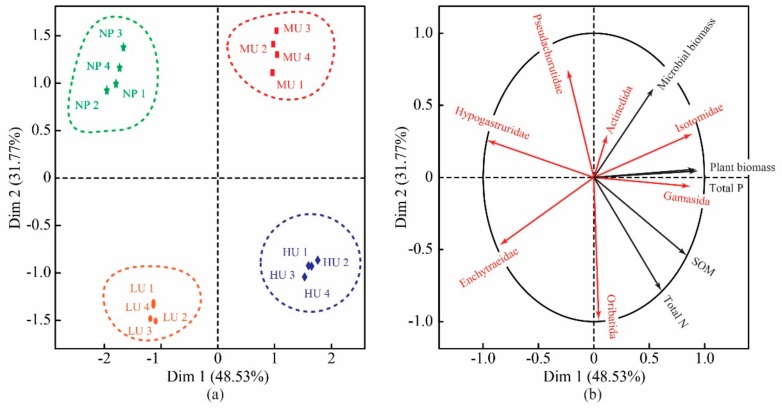
Two-dimensional diagram of multiple factor analysis (MFA). (**a**) Two-dimensional scatter diagram of the MFA performed for the different sampling sites in the alpine tundra of the Changbai Mountains. HU: high level of *D. purpurea* upward expansion; MU: medium level of *D. purpurea* upward expansion; LU: low level of *D. purpurea* upward expansion; NP: native plant habitats. (**b**) Two-dimensional ordination plot of the MFA performed for the entire data set, including environment factors and soil mesofauna data. The percentages appearing next to the axis numbers are the total variation of the data explained by that axis.

A two-dimensional scatter diagram of the multiple factor analysis (MFA) was completed to examine the variations of *D. purpurea* upward expansion (Figure 3). The results show the distribution of the sampling sites within the space formed by the first two-dimensional axis of the MFA, which explains 80.30% of the total variance. The dimension 1 axis of the MFA explains 48.53% of the variances, and the dimension 2 axis explains 31.77%. As illustrated by the scatter diagram, each sampling plot from the HU, MU, LU, and NP (from the positive direction to the negative direction) is located along the dimension 1 axis; thus, the dimension 1 axis indicates the level of upward expansion of *D. purpurea*, in which the positive direction is considered as the greater level of *D. purpurea* upward expansion, while a negative direction indicates the lower level of *D. purpurea* upward expansion (Figure 3a).

A two-dimensional ordination plot of the MFA was constructed to determine the correlations of herbaceous plant upward expansion to the dominant and common taxa of soil mesofauna (Figure 3b). As illustrated by the ordination plot, Isotomidae and Gamasida, as well as Hypogastruridae and Enchytraeidae were positively related to the dimension 1 axis, thus indicating that these taxa were sensitive to herbaceous plants’ upward expansion. Among these, Isotomidae and Gamasida pointed towards the positive direction of the dimension 1 axis, and were thus considered to be positive indicators of *D. purpurea* upward expansion. In contrast, Hypogastruridae and Enchytraeidae pointed toward the negative direction, indicating an ability to negatively respond to the upward expansion of *D. purpurea*. At the same time, Oribatida, Actinedida, and Pseudachorutidae were located almost perpendicular to the dimension 1 axis, indicating that the responses of these soil mesofauna to *D. purpurea*’s upward expansion were relatively slow. In addition, the ordination plot also shows that the soil properties and plant biomass toward the positive direction of the dimension 1 axis, indicating that the *D. purpurea* expansion upwards could promote the contents of the soil properties and plant biomass.

## 3. Discussion

### 3.1. Distribution Patterns of Soil Mesofaunal Communities

In this study, we observed that soil mesofauna in the those *Deyeuxia purpurea* upward expansion treatments (HU, MU, and LU) were all greater than in the native plants habitats (NP), whereas the richness and diversity in the NP were greater than in the high and low upward expansion treatments (HU and LU). This was partially in line with our hypothesis that the upward expansion of *D. purpurea* promotes abundance, richness, and diversity of soil mesofauna (H1). We observed that the *D. purpurea* upward expansion increased the soil properties and plant biomass in the alpine tundra of the Changbai Mountains, which was in agreement with the results of a previous study [15]. In the alpine tundra of the Changbai Mountains, *D. purpurea* originate from the dwarf birch forests [16], where the altitude is relatively low. Previous studies have revealed that, due to the thicker cuticles and a large number of recalcitrant compounds, plants grown at high altitude typically decompose more slowly than those at low altitude [17]. At the same time, the upwardly expanding plants from low altitudes have higher quantities of litter fall than native plants. In light of these reasons, the rates of nutrient release will be accelerated in the alpine tundra of the Changbai Mountains, while the native plants (e.g., *Rhododendron lapponicum* and *Vaccinium uliginosum*) are being replaced by. *D. purpurea*. Consequently, the upward expansion of *D. purpurea* can increase soil fertility and plant biomass in the alpine tundra. It is known that abundant soil fertility and plant biomass can provide substantial amounts of food and comfortable living conditions to soil mesofauna [18,19], and therefore *D. purpurea*’s upward expansion increases soil mesofaunal abundances in the alpine tundra of the Changbai Mountains.

Upwardly expanding plants, which often form dense populations, can directly participate in the competition for resources with native plants, and thereby reduce the abundance and diversity of native plant species or alter the composition of plant communities [20,21]. At the initial stages of plant upward expansion, the native plants are replaced by upward-expanding plants, and the percentages of native plants gradually decline. The disappearance of native plants will alter the nutrient flow, chemical cascade, and chemical communication in the native food chain. As a result, the native taxa of soil mesofauna, which have adapted to the original environment, are gradually disappearing. Consequently, the richness and diversity of soil mesofauna in the LU treatment have become relatively low. At the final stages of plant species, the native plants are almost completely replaced by upward expanding plants, which leads to the diversity of plants being relatively low in the HU treatment. The smaller number of plant species provide relatively unvaried food resources to soil mesofauna. This puts some of the dominant taxa of the soil mesofauna in a great position and reduces the taxa numbers of the rare taxa. Therefore, the HU treatment also exhibited lower richness and diversity of soil mesofauna.

This study observed that richness and diversity of soil mesofauna in the medium upward expansion treatment (MU) were all at the greatest level (Table 1). The intermediate disturbance hypothesis indicates that the intermediate disturbance can promote species diversity in ecological communities, thus permitting invasion and colonization by a greater number of species [22]. Compared with other habitats in the alpine tundra of the Changbai Mountains, a relatively intermediate disturbance was found in the MU habitats. The investigation has shown that plant diversity in the MU habitats is greater than in the herbaceous plant upward-expansion habitats (Table 2), and abundant plant species can complicate the composition of plant litter. In general, multitudinous kinds of plant litter can promote the diversity of soil mesofauna. In addition, some previous studies have demonstrated that mixed species can promote the rates of plant litter decomposition and nutrient release, which creates good soil conditions, and thus many more taxa of soil mesofauna are attracted there to colonize [23]. Therefore, the MU treatment exhibited the greatest richness and diversity of soil mesofauna in the alpine tundra of the Changbai Mountains.

### 3.2. Responses of Soil Mesofauna to the Upward Expansion of Herbaceous Plants 

**Figure 4:**
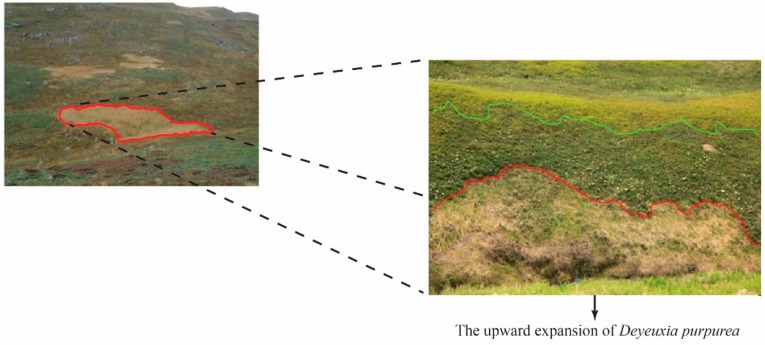
Upward expansion of *Deyeuxia purpurea* in the alpine tundra of the Changbai Mountains.

The Venn diagram and clustering analysis showed that there was a dissimilarity in the soil mesofaunal taxonomic compositions among the different treatments (Figure 1b and Figure 3). These results indicate that the soil mesofaunal taxonomic compositions are affected by *D. purpurea* upwardly expanding in the alpine tundra of the Changbai Mountains. Compared with the *D. purpurea* upward expansion treatments, no unique taxa were collected in the native plant habitats (NP). Formicidae, Juliformia, Japygidae, Cicindelidae, Aphidoidae, Pholcidae, Onychiruidae, and Phloeothripoidae were only collected in the *D. purpurea* upward expansion treatments (HU, MU, and LU), and the majority of these taxa are predatory invertebrates. Previous studies have revealed that the rise of upwardly expanding plants can alter the composition and diversity of native plant communities, with significant consequences that affect the abundance of phytophagous and saprophagous invertebrates [24,25]. The present study found that *D. purpurea* upward expansion increased the abundance of Isotomidae (Figure 4), which consists of phytophagous or saprophagous invertebrates [26]. It is known that abundant numbers of phytophagous and saprophagous invertebrates attract the colonization of greater numbers of predatory invertebrates [27]. Consequently, we conclude that *D. purpurea* upward-expansion treatments will promote the colonization of predatory invertebrates, and that the soil’s mesofaunal guild characteristics will be altered by the upward expansion.

This study found that the different taxa of soil mesofauna respond to the upward expansion of *D. purpurea* in various ways, and this confirms our hypothesis that the different taxa of soil mesofauna respond to herbaceous plant upward expansion at various levels (H2). We observed that Isotomidae and Gamasida responded positively to herbaceous plant upward expansion in the alpine tundra of the Changbai Mountains. Gisin considered that the majority of Isotomidae are part of the Epedaphic (surface-active) and hemiedaphic (within the soil and partly on the surface) Collembola [28]. Previous studies have reported that soil surfaces became progressively wetter and litter layer thickness gradually increased in the alpine tundra of the Changbai Mountains as the upward expansion of herbaceous plants progressed, which established suitable living conditions for epedaphic and hemiedaphic Collembola. As a result, Isotomidae responded positively to herbaceous plant upward expansion. Previous research has shown that most species of Gamasida living in the soil are examples of predatory invertebrates [29]. Due to the rise of phytophagous and saprophagous invertebrates, Gamasida’s abundance was increased, and thus it was considered to be a positive indicator of herbaceous plant upward expansion in the alpine tundra of the Changbai Mountains. In addition, multiple factor analysis (MFA) indicated that Hypogastruridae and Enchytraeidae showed an ability to negatively respond to herbaceous plants’ upward expansion, and the responses of Oribatida, Actinedida, and Pseudachorutidae were relatively slow. The previous research of soil mesofauna has concluded that the life history, nutrition methods, breeding characteristics, and adaptability mechanisms are significantly varied among different taxa [30]. Consequently, the different taxa of soil mesofauna respond to herbaceous plants’ upward expansion to various degrees.

## 4. Materials and Methods

### 4.1. Site Description

In this study, we set up the experimental sites in the alpine tundra of Tianchi volcano (42°02′ N, 128°03′ E), which is located on the western slope of the Changbai Mountains. Tianchi volcano is the one of the most famous volcanoes in the world, and a certain area of alpine tundra can be found from 2000 to 2500 m. The climate in the alpine tundra is the alpine climate of monsoon types in the temperate zonal continent, which is cold, windy, and naturally sunny with intense ultraviolet. The mean annual temperature is about –7 °C, the mean annual wind speed is 11.7 m/s, the mean daily sunshine time is about 100 d, the mean annual total solar radiation is 122.0–122.5 kcal/cm^2^, the active accumulated temperature (≥10 ℃) is 300~500 ℃. The surface is covered with snow more than 6 months per year. The mean precipitation (from June to September) is approximately 800~900 mm. The soil type is Gelisols, and the dominant species of the site were found to be *Vaccinium uliginosum*, *Dryas octopetala, Rhondodendron confertissimum, Pedicularsi verticillate, Bistorta vivipara, Lloydia serotine, Sanguisorba sitchensis*, and *Rhododendron aureum*. Recently, *D. purpurea* has been expanding upwards into the alpine tundra, and has become the dominant species in a partial region of the alpine tundra of the Changbai Mountains (Figure 4).

### 4.2. Sampling Design

To evaluate the responses of soil fauna to low-altitude plants upwardly expanding into the alpine tundra of the Changbai Mountains, four treatments were prepared. The treatments consisted of high upward expansion level (HU; herbaceous coverage: 90%), medium upward expansion level (MU; herbaceous coverage: 60%), low upward expansion level (LU; herbaceous coverage: 30%), and native plant habitats (NP; herbaceous coverage 0%). The geographical information and ecological parameters of each are shown in Table 3.

Due to the extremely cold and windy climate in other seasons, we collected the samples in summer (July) of 2018. Four replicated stands of each treatment were randomly established on the alpine tundra at 500 m intervals. In each stand, we setup a plot (400 m^2^), and four replicated subplots (1 m^2^) were randomly established at 5 m intervals. The aspect of each plot was towards the west, and the gradient was less than 20°. To collect epedaphic and hemiedaphic soil mesofauna, litter samples (100 cm^2^) were taken from each subplot. At the same time, soil samples (area: 100 cm^2^; depth: 10 cm) were collected to extract euedaphic-living soil mesofauna. A total of 128 samples (4 treatments × 4 replicated stands × 1 plot × 4 subplots × 2 layers) were collected. All of litter and soil samples were then taken back to the laboratory. Tullgren funnel extractors (Burkard Mfg. Co., Ltd., Hertfordshire, UK) were used to extract soil mesofauna in four days (96 h). After extracting, all soil mesofauna were saved into vials in 75% alcohol. The soil mesofauna were counted and identified using an OLYMPUS SZX16 stereoscopic microscope and an OLYMPUS CX41 biomicroscope (Olympus Co., Tokyo, Japan). In this study, the majority of soil mesofauna were identified at the family level, and mites were identified at the suborder level [31].

To determine soil properties, an additional 64 soil samples (4 treatment × 4 replicated stands × 1 plot × 4 subplots) were collected by soil augers beside the sample site for the soil mesofauna in each subplot. The organic matter was digested by K_2_Cr_2_O_7_-H_2_SO_4_ and examined using FeSO_4_ titration; the total nitrogen (N) levels were digested by H_2_SO_4_ and K_2_SO_4_–CuSO_4_∙5H_2_O–Se. The total P samples were digested by H_2_SO_4_ and HClO_4_ and examined using a SmartChem140 analyzer (Senesia SRL, Milan, Italy) [32]. The soil microbial PLFAs were measured according to previously published methods [33]. 

### 4.3. Data Analysis

Data was calculated from the pooled data of two layers from each sampling site. Generalized linear models (GLMs) were conducted to test the differences in the soil mesofaunal abundance (individuals per m^−2^; collected individuals × 100), Shannon–Wiener index ($H\prime =-\overset{s}{\underset{i=1}{\sum }}P_{i}lnP_{i}$, where *S* is the number of species and *Pi* is the ratio of individuals to the total collected individuals in species *i* for each treatment) [34], soil properties, and plant biomass, using SPSS 22 (SPSS Inc., Chicago, IL, USA). A Venn diagram was illustrated to evaluate the effect of *D. purpurea*’s upward expansion on the composition of the soil mesofaunal communities. The endemic taxa of each treatment were determined, and then the Venn diagram was created using a “draw-quintuple-Venn” function, available in a VennDiagram R package [35].

A cluster analysis (unweighted pair group method with a mathematical mean algorithm, or UPGMA) was conducted to analyze the similarities in the different soil mesofaunal communities within four treatments. The “hclust” function available in the stats R package was executed to create the UPGMA cluster [36]. In order to demonstrate the distribution patterns of the different soil mesofaunal taxa, a heat map of the hierarchical clustering was illustrated by using a “pheatmap” R package [37], along with the “vegemite” function available in the vegan R package [38].

Multiple factor analysis (MFA), which seeks common structures among data matrices, was conducted on the data sets of abundance of soil mesofauna, soil organic matter, soil PLEAs, plant biomass, total N, and total P, so as to test the relationship between soil mesofauna and environmental changes caused by the upward expansion of *D. purpurea* [39]. Prior to executing the MFA, the data sets of the soil mesofauna were transformed by the “Hellinger transformation” [40,41]. The MFA was performed using the “MFA” function, available in the FactoMineR R package [42]. 

## 5. Conclusions

In summary, it is shown here that the abundance of soil mesofauna can indicate the levels of upward expansion of *Deyeuxia purpurea*, and that the amount of soil mesofauna increased with the rise of this upward expansion. In addition, the taxonomic composition of soil mesofauna varied with the different levels of herbaceous plant upward expansion in the alpine tundra of the Changbai Mountains. *Deyeuxia purpurea* upward expansion promoted the colonization of predatory invertebrates, and the soil mesofaunal guild characteristics were altered by the upward expansion. The different taxa of soil mesofauna responded to herbaceous plants’ upward expansion to various degrees. Isotomidae and Gamasida responded positively to herbaceous plant upward expansion and were considered to be a positive indicator of it. Hypogastruridae and Enchytraeidae responded relatively negatively, while Oribatida, Actinedida and Pseudachorutidae responded sluggishly. The findings of this study have implications for the relationship between herbaceous plant upward expansion and soil mesofauna, and can provide some help in developing biodiversity guidelines for the protection of alpine tundra.

## Figures and Tables

**Table 1:** Abundance (individuals/m^2^), richness (taxa number), and Shannon–Wiener index of the soil mesofaunal communities in the different treatments.

	Treatment			
	HU	MU	LU	NP
Abundance (individuals/m^2^)	15,625 ± 566 a	14,450 ± 717 a	12,375 ± 657 b	9675 ± 705 c
Richness (taxa number)	17	20	16	20
Shannon–Wiener Index	1.82 ± 0.09 b	1.95 ± 0.12 a	1.83 ± 0.11 b	1.86 ± 0.08 b

Lower case letters (a, b, and c) indicate the significant differences in each treatment within the same abundance and Shannon–Wiener index at the *p* < 0.05 level. HU: high level of *D. purpurea* upward expansion; MU: medium level of *D. purpurea* upward expansion; LU: low level of *D. purpurea* upward expansion; NP: native plant habitats.

**Table 2:** Soil properties and plant biomass in the different treatments (mean ± SE). HU: high level of *D. purpurea* upward expansion; MU: medium level of *D. purpurea* upward expansion; LU: low level of *D. purpurea* upward expansion; NP: native plant habitats.

	Levels of *Deyeuxia purpurea* Expansion Upwards			
	HU	MU	LU	NP
Soil organic matter (g/kg)	0.21 ± 0.09 a	0.27 ± 0.23 a	0.12 ± 0.04 b	0.20 ± 0.08 a
Soil total N (g/kg)	7.72 ± 0.42 b	9.74 ± 0.48 a	5.98 ± 0.09 c	8.78 ± 0.04 ab
Soil total P (g/kg)	0.51 ± 0.05 a	0.58 ± 0.01 a	0.38 ± 0.01 b	0.35 ± 0.02 b
Soil PLFA contents (nmol/g)	31.52 ± 3.93 a	8.99 ± 0.79 bc	8.58 ± 0.34 c	8.40 ± 0.38 c
Plant biomass (kg/m^2^)	1.27 ± 0.11 b	1.63 ± 0.09 a	0.69 ± 0.05 c	0.50 ± 0.01 c

The least significant difference (LSD) was used to compare the means. Values with different letters within a row show means with significant differences at *p* < 0.05.

**Table 3:** Geographical information and ecological parameters of each treatment. HU: high level of *D. purpurea* upward expansion; MU: medium level of *D. purpurea* upward expansion; LU: low level of *D. purpurea* upward expansion; NP: native plant habitats.

		Treatments		
	HU	MU	LU	NP
Location	42°24′8.39″ N, 128°5′47.45″ E	42°22′22.67″ N, 128°5′52.77″ E	42°23′57.55″ N, 128°8′21.56″ E	42°24′50.69″ N, 28°8′32.77″ E
Altitude (m)	2251	2253	2250	2252
Shrub coverage (%)	8	15	20	30
Herb coverage (%)	92	85	80	70
*Deyeuxia purpurea* coverage (%)	90	60	30	0
Years of upward expansion (*a*)	3	2	1	-
Plant diversity	0.91	1.71	1.14	1.36
Plant evenness	0.62	0.38	0.7	0.52
Plant richness	3.56	2.14	1.28	1.74
Plant biomass (kg/m^2^)	1.27	1.63	0.68	0.50
Thickness of litter layers (cm)	5.01	4.96	1.68	3.89

## Supplementary Materials

The following are available online at https://www.mdpi.com/2223-7747/8/12/615/s1, Table S1: Abundance (ind. m^−2^) of soil mesofauna in the different treatments.
