# Supplementary material for: Soil Mesofauna Respond to the Upward Expansion of Deyeuxia purpurea in the Alpine Tundra of the Changbai Mountains, China
Source: Plants (Basel). 2019 Dec 17;8(12):615. doi: 10.3390/plants8120615 (PMC6963277; doi:10.3390/plants8120615)
Supplement: Supplementary file 1 [file plants-08-00615-s001.pdf]

Supplementary Material Table S1 Abundance (ind. m^{-2}) of soil mesofauna in the different treatments. HU, high level of *Deyeuxia purpurea upward expansion*; MU, medium level of *D. purpurea upward expansion*; LU, low level of *D. purpurea upward expansion*; NP, native plant habitats.

Orders	Taxa	Levels of *Deyeuxia purpurea* upward expansion				Total	%
		HE	ME	LE	NP		
Tubificida	Enchytraeidae	600	1100	1000	1600	4300	2.06
Lithobiomorpha	Lithobiidae	0	500	0	200	700	0.34
Juliformia	Juliformia	100	0	0	0	100	0.05
Acariformes	Oribatida	7200	19500	5300	17000	49000	23.50
	Actinedida	9200	6100	5700	4900	25900	12.42
Parasitiformes	Gamasida	14100	9100	2300	4300	29800	14.29
Araneae	Anyphaenidae	0	200	0	100	300	0.14
	Pholcidae	0	100	0	0	100	0.05
Diplura	Japygidae	100	0	0	0	100	0.05
Poduromorpha	Onychiruidae	0	0	100	0	100	0.05
	Hypogastruridae	3900	1000	7200	2700	14800	7.10
	Pseudachorutidae	3800	2800	7600	500	14700	7.05
	Neanuridae	0	0	100	100	200	0.10
Entomobryomorpha	Tomoceridae	0	1100	0	700	1800	0.86
	Entomobryidae	800	400	500	300	2000	0.96
	Isotomidae	21400	12900	18700	4900	57900	27.77
Symphypleona	Sminthuridae	100	300	0	300	700	0.34
Thysanoptera	Phloeothripoidea	0	0	100	0	100	0.05
Homoptera	Aphidoidea	0	200	0	0	200	0.10
Hymenoptera	Formicidae	400	0	0	0	400	0.19
Diptera	Brachycera	100	100	0	100	300	0.14
	Nematocera	200	0	200	0	400	0.19
	Cyclorrhapha	100	0	200	100	400	0.19
Coleoptera	Carabidae	300	100	0	300	700	0.34
	Staphyinidae	0	1300	200	300	1800	0.86
	Elateridae	0	0	100	100	200	0.10
	Scarabaeidae	0	0	200	100	300	0.14
	Cantharidae	100	300	0	0	400	0.19
	Cicindelidae	0	500	0	0	500	0.24
	Cleridae	0	200	0	100	300	0.14
